# Therapeutic Applications of Essential Oils from Native and Cultivated Ecuadorian Plants: Cutaneous Candidiasis and Dermal Anti-Inflammatory Activity

**DOI:** 10.3390/molecules28155903

**Published:** 2023-08-05

**Authors:** Lilian Sosa, Lupe Carolina Espinoza, Eduardo Valarezo, Núria Bozal, Ana Calpena, María-José Fábrega, Laura Baldomà, María Rincón, Mireia Mallandrich

**Affiliations:** 1 Microbiological Research Institute (IIM), National Autonomous University of Honduras (UNAH), Tegucigalpa 11101, Honduras; lilian.sosa@unah.edu.hn; 2 Research Institute of Applied Sciences and Technology, National Autonomous University of Honduras (UNAH), Tegucigalpa 11101, Honduras; 3 Departamento de Química, Universidad Técnica Particular de Loja, Loja 1101608, Ecuador; lcespinoza@utpl.edu.ec (L.C.E.); bevalarezo@utpl.edu.ec (E.V.); 4 Institut de Nanociència i Nanotecnologia (IN2UB), Universitat de Barcelona (UB), 08028 Barcelona, Spain; anacalpena@ub.edu; 5 Departament de Biologia, Sanitat i Medi Ambient, Facultat de Farmàcia i Ciències de l’Alimentació, Universitat de Barcelona (UB), 08028 Barcelona, Spain; nuriabozaldefebrer@ub.edu; 6 Departament Farmàcia, Tecnologia Farmacèutica, i Físicoquímica, Facultat de Farmàcia i Ciències de l’Alimentació, Universitat de Barcelona (UB), 08028 Barcelona, Spain; 7 Department of Experimental and Health Sciences, Parc of Biomedical Research of Barcelona, Pompeu Fabra University, 08003 Barcelona, Spain; mjfabrega.f@gmail.com; 8 Departament de Bioquímica i Fisiologia, Facultat de Farmàcia i Ciències de l’Alimentació, Universitat de Barcelona (UB), 08028 Barcelona, Spain; lbaldoma@ub.edu; 9 Departament de Ciència de Materials i Química Física, Facultat de Química, Universitat de Barcelona (UB), 08028 Barcelona, Spain; m.rincon@ub.edu

**Keywords:** *Candida albicans*, *Candida glabrata*, *Candida parapsilosis*, skin inflammation, essential oil, *Bursera graveolens*, *Dacryodes peruviana*, *Mespilodaphne quixos*, *Melaleuca armillaris*

## Abstract

Essential oils are a complex mixture of aromatic substances whose pharmacological actions, including antimicrobial, antioxidant, anticancer, and anti-inflammatory activities, have been widely reported. This study aimed to evaluate the anti-*Candida* and dermal anti-inflammatory activity of essential oils from native and cultivated Ecuadorian plants. Essential oils from *Bursera graveolens*, *Dacryodes peruviana*, *Mespilodaphne quixos*, and *Melaleuca armillaris* were isolated by hydrodistillation and were characterized physically and chemically. Its tolerance was analyzed by in vitro and in vivo studies. The antifungal activity was studied against *Candida albicans*, *Candida glabrata*, and *Candida parapsilosis*, whereas the anti-inflammatory effect was evaluated by a mouse ear edema model. The main compounds were limonene, α-phellandrene, (*E*)-methyl cinnamate, and 1,8-cineole, respectively. All essential oils showed high tolerability for skin application, antifungal activity against the three *Candida* strains, and anti-inflammatory efficacy by decreasing edema and overexpression of pro-inflammatory cytokines. *Dacryodes peruviana* essential oil showed the highest antifungal activity. On the other hand, *Dacryodes peruviana* and *Melaleuca armillaris* showed the greatest anti-inflammatory potential, decreasing edema by 53.3% and 65.25%, respectively, and inhibiting the overexpression of TNF-α, IL-8, IL-17A, and IL-23. The results suggest that these essential oils could be used as alternative therapies in the treatment of both cutaneous candidiasis and dermal inflammation.

## 1. Introduction

Fungal infections represent a public health problem affecting almost one billion people worldwide. These infections appear in different anatomical sites such as the skin, nails, scalp, and vagina [1]. The skin is the largest organ in the body and can suffer from fungal infections such as candidiasis. Cutaneous candidiasis is a superficial form of mycosis caused by the proliferation of *Candida* spp. fungi in the skin, especially in intertriginous areas causing itching, erosion, inflammation, and pustules [2,3].

*Candida albicans* is the species that produces most of the cases of candidiasis. However, in America and Europe (except for Spain), the cases have increased due to the appearance of *Candida glabrata*, while in South America, Japan, and Spain, *Candida parapsilosis* has been the main cause of candidiasis [4]. The clinical importance of non-albicans strains lies in infections caused by strains such as *Candida glabrata*. The incidence of this strain is high in patients with acquired immunodeficiency syndrome (AIDS), cancer, and diabetes and in those who use medical devices. Likewise, it has been reported as a fungus resistant to azoles (first-line treatment), with a faster propagation than *Candida albicans* and a higher mortality rate [5,6]. In the case of infections caused by *Candida parapsilosis*, this strain is responsible for a third of neonatal *Candida* infections reaching a mortality rate of approximately 10%. In addition, patients who require prolonged use of a central venous catheter are also at increased risk of infection due to the innate ability of this fungus to adhere to prosthetic surfaces and implanted medical devices due to the biofilm they produce. The structure of this biofilm shows high variability in different clinical isolates of *Candida parapsilosis* [7].

The treatments used for dermal candidiasis consist of the use of topical azoles that inhibit the biosynthesis of the ergosterol component of the cell membrane and some macrolides such as nystatin and amphotericin B, which bind to the ergosterol of the membrane to induce porosity and cause cell death through leakage of cytosolic components [8]. Likewise, drugs such as caspofungin have been reported in the literature as possible therapeutic alternatives. This drug inhibits fungal cell membrane formation by disrupting the synthesis of the structural component 1,3-β-d-glucan [9]. Despite advances in antifungal treatments, many patients frequently experience complications related to hepatoxicity and increased fungal resistance to conventional drugs [10].

Inflammation is the response of an organism to tissue damage caused by a foreign agent that can be physical, chemical, or biological [11]. When an inflammatory process is generated, macrophages are activated, which produce pro-inflammatory mediators such as prostaglandins, cyclooxygenases, reactive oxygen species, and cytokines [12]. Tumor necrosis factor alpha (TNF-α), interleukin (IL)-8, IL-17A, and IL-23 are cytokines secreted rapidly in response to an inflammatory process. An inappropriate or excessive inflammatory response in the skin can trigger chronic inflammation causing skin disorders such as dermatitis, psoriasis, and rosacea [13]. TNF-α is produced during acute inflammation by macrophages/monocytes, and it induces the expression of pro-inflammatory mediators such as IL-6, IL-8, and IL-1β [14]. IL-8 is a member of the chemokine family that attracts neutrophils, basophils, and T-cells to sites of inflammation and is released from different cells, including monocytes, macrophages, neutrophils, fibroblasts, endothelial cells, and keratinocytes [15]. IL-17A is produced by a subset of activated CD4 T cells, and it is associated with allergic processes and autoimmune diseases including psoriasis, and turn, increases the levels of IL-6 and IL-8 [16]. IL-23 is a dimeric cytokine that promotes the development and differentiation of effector Th17 cells, and thus, it has been implicated in chronic immune-inflammatory disorders such as arthritis, colitis, gastritis, and psoriasis [17].

Nonsteroidal anti-inflammatory drugs (NSAIDs), including diclofenac, fenoprofen, ibuprofen, ketoprofen, ketorolac, and naproxen, are one of the most widely used groups of drugs for treating inflammation, pain, and fever. However, these drugs have been associated with adverse gastrointestinal, renal, hepatic, and skin reactions [18,19].

In the search for effective compounds and alternative therapies to treat cutaneous candidiasis and dermal inflammation, natural products, especially essential oils, stand out as promising candidates in antifungal and anti-inflammatory treatments [20].

An essential oil (EO) is a complex mixture of aromatic substances isolated from diverse types of aromatic plants. Various scientific studies have reported numerous pharmacological actions for essential oils, including antimicrobial, antioxidant, anticancer, and anti-inflammatory properties [21]. Essential oils are used in the pharmaceutical, cosmetic, and food industries [22,23].

*Burseraceae* is a family of 18 genera and about 650 species [24]. A total of 40 species of this family have been reported in the megadiverse country of Ecuador [25]. Some studies show that the fruits of Burseraceae species contain a high yield (2–5%) of essential oil [26]. *Bursera graveolens* (Kunth) Triana and Planch is a shrub or tree commonly known as “palo santo” that grows in dry forests of tropical America and on the Pacific coast of South America, especially in Colombia, Costa Rica, El Salvador, Ecuador, Guatemala, Honduras, Mexico, Nicaragua, and Peru. In Ecuador, *B. graveolens* can mainly be found in the provinces of Galapagos, Guayas, Imbabura, Loja, and Manabí [25]. Essential oil of *B. graveolens* can be found in its leaves, trunk, and fruits and has shown acaricidal, antimicrobial, antioxidant, and repellent activities [26,27].

*Dacryodes peruviana* (Loes.) H.J. Lam (Burseraceae) is a tree of the Burseraceae family known as “copal”, which grows in the humid forests of Colombia, Peru, Ecuador, and Venezuela [28]. In Ecuador, *D. peruviana* has the status of native and is widely distributed in the Amazonian and Andean Ecuadorian regions between 0 and 2500 m above sea level [25]. The essential oil of copal fruits has shown antimicrobial and repellent activity [29].

*Lauraceae* comprises approximately 2978 accepted species in nearly 68 genera worldwide [30]. Within this family, the *Mespilodaphne* is a small genus with 15 individuals [31]. *Mespilodaphne quixos* (Lam.) Rohwer (Syn. *Ocotea quixos* (Lam.,) Kosterm, accepted name *Mespilodaphne quixos* and validated name *Ocotea quixos*) [32] is an Ecuadorian native and cultivated aromatic species, widely distributed in the Andean and Amazonian regions between 0 and 1000 m above sea level [33]. The species is commonly known as “ishpingo” or “canela” because it smells similar to cinnamon (“canela” in Spanish). Essential oil of this species has been isolated from its leaves, trunk, fruits, and fruit calices and has been shown to have antimicrobial, anti-inflammatory, antioxidant, antithrombotic, and larvicidal activities [34,35,36,37,38].

*Myrtaceae* is a family of 129 genera and 6233 species distributed in tropical regions worldwide. *Myrtaceae* is a family of evergreen trees or shrubs rich in essential oils [39]. *Melaleuca armillaris* (Sol. ex Gaertn.) Sm. is a species of the *Myrtaceae* family cultivated in Ecuador known by the common name of bracelet honey myrtle or “árbol de papel”. The essential oil of the leaves of *M. armillaris* has shown antimalarial, antioxidant, anticancer, antimicrobial, and phytotoxic activities [40,41].

Based on these remarkable findings, the purpose of this study was to evaluate the therapeutic applications of essential oils isolated from native and cultivated plants of Ecuador in the treatment of cutaneous candidiasis and dermal inflammation, as well as analyze their skin tolerance, to provide effective and safe alternatives in the treatment of these two pathologies.

## 2. Results

### 2.1. Isolation and Physical Properties of Essential Oil

The yield in essential oil and physical properties density, refractive index, and optical activity for the four essential oils used are shown in Table 1. The physical properties of an essential oil are used as a quality parameter. The yield of the fruits, in general, is higher than that of the leaves of a species. According to the classification made by Molares et al. in 2009, the yield of *Melaleuca armillaris* is intermediate (values between 5 mL/kg and 10 mL/kg), and the yields of the other species are high (values greater than 10 mL/kg) [42]. The yield, together with the biomass availability of a species, provides an idea of the amount of essential oil available to scale a project, which makes the essential oil of these species suitable for industrial applications.

### 2.2. Essential Oil Compounds Identification

The identification of volatile compounds contained in *Bursera graveolens*, *Dacryodes peruviana*, *Ocotea quixos* (Accepted name *Mespilodaphne quixos*), and *Melaleuca armillaris* essential oils was carried out using gas chromatography equipped with a flame ionization detector and gas chromatography coupled to a mass spectrometer detector using capillary nonpolar column DB-5Ms. The compounds were classified into six groups: monoterpene hydrocarbons (MH), oxygenated monoterpene (OM), sesquiterpene hydrocarbons (SH), oxygenated sesquiterpene (OS), phenylpropanoids, and other compounds (OC) (Table 2). All essential oil compounds from *Dacryodes peruviana* and most essential oil compounds from *Bursera graveolens* belong to the MH group. The main compounds were limonene, α-phellandrene, (*E*)-methyl cinnamate, and 1,8-cineole in the essential oils of *Bursera graveolens*, *Dacryodes peruviana*, *Mespilodaphne quixos*, and *Melaleuca armillaris*, respectively.

### 2.3. Cytotoxicity Studies by Methylthiazolyldiphenyl-Tetrazolium Bromide (MTT) Method

**Figure 1:**
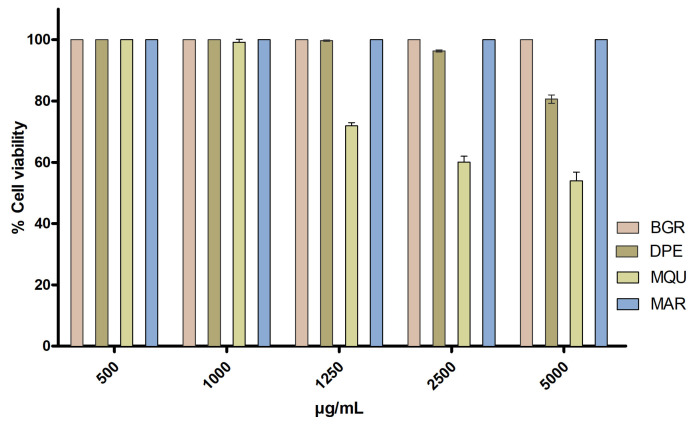
Cytotoxicity of HaCaT cell line exposed to essential oils from *Bursera graveolens* (BGR)*, Dacryodes peruviana* (DPE), *Mespilodaphne quixos* (MQU), and *Melaleuca armillaris* (MAR).

Cell viability of human keratinocytes HaCaT exposed at the different concentrations of the essential oils studied was evaluated by MTT cytotoxicity assay. After 24 h of incubation, it was observed that the essential oils from *Bursera graveolens*, *Dacryodes peruviana*, and *Melaleuca armillaris* at the assayed concentrations from 500 to 5000 µg/mL did not affect cell viability, which was greater to 80% whereas the essential oil from *Mespilodaphne quixos* (MQU) showed cell viability greater to 80% from 500 to 1000 µg/mL (Figure 1). Therefore, these results suggest that the essential oils do not generate toxicity in the keratinocytes at these concentrations.

### 2.4. In Vivo Tolerance Studies by Evaluation of Transepidermal Water Loss (TEWL)

**Figure 2:**
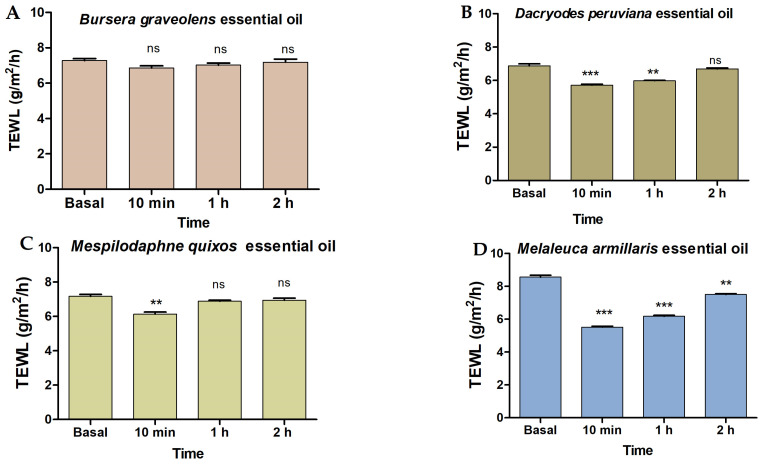
Tolerance studies by transepidermal water loss (TEWL): (**A**) *Bursera graveolens* essential oil; (**B**) *Dacryodes peruviana* essential oil; (**C**) *Mespilodaphne quixos essential oil*; (**D**) *Melaleuca armillaris* essential oil. Statistically significant differences: ** = *p* < 0.01; *** = *p* < 0.001; ns = not significant.

Figure 2A shows that *Bursera graveolens* essential oil did not cause significant differences in TEWL value after topical application on the ventral area of the forearm of the volunteers compared with the basal state, whereas *Dacryodes peruviana*, *Mespilodaphne quixos*, and *Melaleuca armillaris* essential oils significantly reduced the TEWL values after 10 min of topical application (Figure 2B–D). However, a tendency to return to the basal states was observed after 2 h of the experiment.

### 2.5. Efficacy Studies: Antifungal Activity

The Minimum Inhibitory Concentrations (MICs) of essential oil from *Bursera graveolens*, *Dacryodes peruviana*, *Mespilodaphne quixos*, and *Melaleuca armillaris* are shown in Table 3. All the essential oils presented antifungal activity against the three *Candida* strains. *Dacryodes peruviana* essential oil showed the lowest MIC value, and therefore it has greater antifungal activity. On the contrary, the essential oil with minor efficacy was *Melaleuca armillaris* essential oil.

### 2.6. In Vivo Anti-Inflammatory Activity

#### 2.6.1. Arachidonic Acid (AA)-Induced Mouse Ear Edema

**Figure 3:**
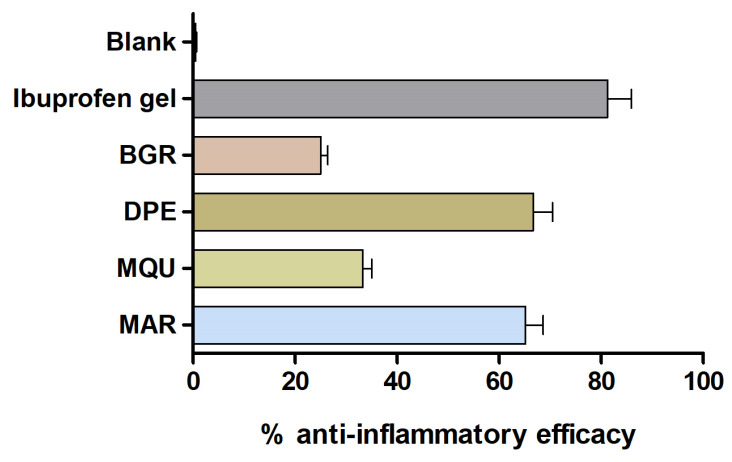
Anti-inflammatory efficacy after AA-induced mouse ear edema. Mean ± SD (*n* = 3). Ibuprofen gel = treatment with reference drug; Blank = Transcutol P:water; treatment with *Bursera graveolens* (BGR), *Dacryodes peruviana* (DPE), *Mespilodaphne quixos* (MQU), and *Melaleuca armillaris* (MAR).

Topical application of AA on the mice’s ears caused immediate erythema followed by the development of edema represented by the increase in the ear thickness as a result of the inflammatory process. However, this parameter decreased notably after topical treatment with the different essential oils tested mainly with *Dacryodes peruviana* (DPE) and *Melaleuca armillaris* (MAR), which decreased edema by 53.3% and 65.25%, respectively, although without reaching the efficacy of the reference drug (ibuprofen) that inhibits edema by 81.25%. The blank solution did not cause changes in the inflammatory process induced by AA (Figure 3).

#### 2.6.2. Pro-Inflammatory Cytokines Determination

**Figure 4:**
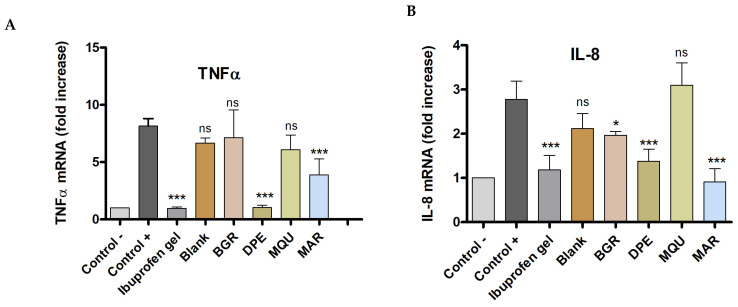

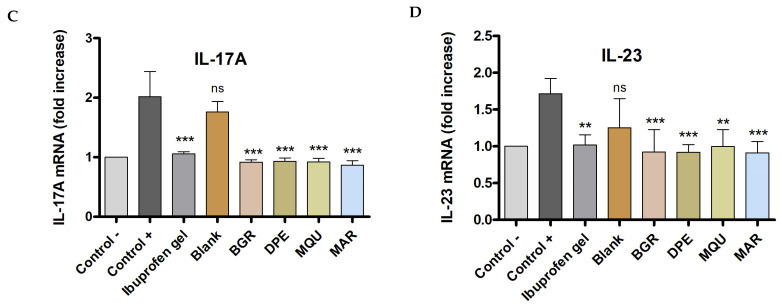
Relative expression of pro-inflammatory cytokines measured by quantitative reverse transcription polymerase chain reaction: (**A**) TNFα = Tumor necrosis factor-alpha; (**B**) IL–8 = interleukin–8; (**C**) IL–17A = interleukin–17A; (**D**) IL-23 = interleukin–23. Control − = negative control; Control + = positive control; Ibuprofen gel = treatment with reference drug; Blank = Transcutol P:water; treatment with *Bursera graveolens* (BGR), *Dacryodes peruviana* (DPE), *Mespilodaphne quixos* (MQU) and *Melaleuca armillaris* (MAR). Statistically significant differences: * = *p* < 0.05; ** = *p* < 0.01; *** = *p* < 0.001; ns = not significant.

The positive control showed a significant increase in the expression of pro-inflammatory cytokines TNF-α, IL-8, IL-17A, and IL-23 by the inflammation triggered by skin application of AA. When compared with the positive control, topical treatment with *Bursera graveolens* essential oil significantly decreased the expression of IL-8, IL-17A, and IL-23, whereas *Mespilodaphne quixos* essential oil reduced the expression of IL-17A and IL-23. Essential oils from *Dacryodes peruviana* (DPE) and *Melaleuca armillaris* (MAR) significantly reduced the expression of all pro-inflammatory cytokines studied TNF-α, IL-8, IL-17A, and IL-23. These results suggest that these essential oils could regulate the inflammatory processes and serve as a treatment or adjuvant in anti-inflammatory therapies (Figure 4).

## 3. Discussion

Essential oils are a complex mixture of aromatic substances, which have shown several pharmacological properties thanks to their various components [44,45]. The uses attributed to essential oils include antiseptic, irritant, spasmolytic, sedative, anti-inflammatory, and antimicrobial potential [2,44,46]. The antimicrobial activity is of great interest because of the phenomenon of resistance to conventional antimicrobial drugs [47]. Specifically, in the present study, the essential oils obtained from four different plants: *Bursera graveolens*, *Dacryodes peruviana*, *Mespilodaphne quixos*, and *Melaleuca armillaris* were studied physically, chemically, and microbiologically to establish their possible pharmacological use in the treatment of both cutaneous candidiasis and dermal inflammation. The yields of essential oils obtained from these species are suitable for various industrial applications, including pharmacological use. High yields were observed for the essential oils of *Bursera graveolens, Dacryodes peruviana*, and *Mespilodaphne quixos* with values greater than 10 mL/kg. In comparison, the yield for the essential oil of *Melaleuca armillaris* was intermediate, with values between 5 mL/kg and 10 mL/kg [42].

Chemical characterization showed that the main compounds for the essential oils of *Bursera graveolens*, *Dacryodes peruviana*, *Mespilodaphne quixos*, and *Melaleuca armillaris* are limonene, α-phellandrene, (*E*)-methyl cinnamate, and 1,8-cineole, respectively. In previous studies carried out on the essential oils of these species, similar compounds were found, although with different percentages. For example, limonene was the main compound in essential oil isolated from the fruits of *Bursera graveolens*, with percentages from 43.6% [26] to 49.89% [27]. In the essential oil of fruits *of Dacryodes peruviana*, α-phellandrene (52.4%) and limonene (22.5%) were determined as the major compounds [46]. In essential oil extracted from the leaves of *Mespilodaphne quixos*, the main components were β-caryophyllene (15.1%) and cinnamyl acetate (11.4%) [48]; however, in a study on the variability of the chemical composition of this essential oil with the amount of shade, type of soil and altitude, it was determined that there is a great variation in the percentages of the major compounds (*E*)-cinnamyl acetate (5.96–41.65%), (*E*)-methyl cinnamate (0.38–37.91%), and trans-caryophyllene (8.77–37.02%) [49]. In the essential oil extracted from leaves of *Melaleuca armillaris*, the 1,8-cineole was determined as the main component with percentages from 68.9% [50] to 85.8% [40].

To evaluate the tolerability of these essential oils for topical application on the skin, in vitro and in vivo were performed. In vitro models allowed for screening the toxicity of substances prior to in vivo assessment. Cell lines are highly used for toxicity screening in a living system because they are generally easy to cultivate, fast to grow, and sensitive to toxic irritation [51]. In this study, the results confirmed that the essential oils at the tested dilutions do not induce relevant cytotoxic effects, and thus they have high biocompatibility with human keratinocytes. This result was confirmed by further studies in volunteers by evaluation of the transepidermal water loss (TEWL) parameter, which allows for the evaluation of the skin barrier’s integrity after exposure to physical or chemical agents. TEWL is a biomechanical parameter of the skin that indicates water loss across the stratum corneum. Its determination is a noninvasive in vivo valuable method in dermatology to detect disturbances of the skin barrier in early stages, even before they become visible. In damaged skin, TEWL increases, and subsequently, the hydration declines, causing cracks and fissures in the stratum corneum [51]. As a general result, the essential oils studied did not cause skin irritation in the volunteers indicating biocompatibility and suitability for human use. *Bursera graveolens* essential oil did not cause significant differences in TEWL value after skin application compared with the basal state, whereas a significant reduction in TEWL after 10 min of skin application of *Dacryodes peruviana*, *Mespilodaphne quixos*, and *Melaleuca armillaris* essential oils was observed but with a tendency to return to the basal states after 2 h of the experiment. These results suggest an enhancement of the hydration level of stratum corneum, probably due to the occlusive effect of these essential oils.

Regarding antifungal activity, all the essential oils were effective against *Candida albicans, Candida glabrata*, and *Candida parapsilosis*. Essential oils from *Bursera morelensis* belonging to the same family as *Bursera graveolens*, have shown an antifungal effect, specifically on *Candida albicans* [52]. In addition, previous studies have reported the antifungal activity of *Bursera graveolens* essential oil against *Candida albicans* [53]. Likewise, it has been shown that this essential oil could enhance the antifungal effect of drugs such as amphotericin B, improving its activity against various strains of *Candida* [2]. *Dacryodes peruviana* essential oil showed the lowest MIC value, and therefore it has greater antifungal activity. The antifungal activity of *Bursera graveolens* and *Dacryodes peruviana* essential oil could be due to its rich content in limonene and α-phellandrene. Specifically, *Bursera graveolens* essential oil showed the highest content in limonene (49.4 ± 2.2%) and *Dacryodes peruviana* essential oil showed the highest content of α-phellandrene (50.3 ± 3.3%). Studies have shown that limonene exhibits various biological effects, including excellent anti-*Candida* activity [54]. It is considered that the antifungal activity of this compound against *Candida* could be due to failure of ion transshipment and ATP generation in the membrane; disruption of intracellular ion homeostasis; and extensive leakage of intracellular substance caused by potential, permeability, and integrity of the cell membrane. In addition, limonene also leads to suppression or intracellular metabolic disorders in *Candida* [55,56]. On the other hand, the activity exerted by α-phellandrene could be because this molecule disrupts the integrity of the fungal cell membrane, causing leakage of potassium ions and cell constituents, which would trigger an increase in total lipid content, extracellular pH, and membrane permeability, causing the death of the fungus [57]. Additionally, it has been shown that the biotransformation of α-phellandrene to 5-p-menthene-1,2-diol would generate antifungal activity against *Candida* [58]. The effect of *Dacryodes peruviana* essential oil against candida is interesting since its antifungal effect has been demonstrated, but only against *Trichophyton rubrum* and *Trichophyton mentagrophytes* [29].

The antifungal effect of *Mespilodaphne quixos* essential oil has been reported in previous studies against *Candida albicans* and *Saccharomyces cerevisiae* [59]. This efficacy could be due to the synergy of the components of this essential oil, as well as its high content of (*E*)-methyl cinnamate (19.3 ± 1.3%) and trans-caryophyllene (15.8 ± 1.8%). Both components have shown an effect on *Candida* species [35,60]. However, their mechanism of action is not fully elucidated, and more studies are necessary to establish the antifungal mechanism exerted against these pathogenic fungi [61]. Finally, *Melaleuca armillaris* essential oil showed higher MIC values than those found for *Dacryodes peruviana* and *Mespilodaphne quixos* essential oil and similar to those found for *Bursera graveolens* essential oil. However, an antifungal effect was observed against three *Candida* strains tested. Previous studies have reported the anti-*Candida* effect of essential oils obtained from various species of *Melaleuca* spp. [62]. The main component found in *Melaleuca armillaris* essential oil was 1,8-cineole (83.4 ± 2.5%). This component has demonstrated its antifungal effect against *Candida albicans*, either alone or in combination with other drugs [63,64,65]. More in-depth studies are necessary to determine the antifungal mechanism of action of this essential oil against *Candida*. Together, these results suggest that the four essential oils studied could be used as active antifungal components or enhancers of conventional drugs in treating cutaneous candidiasis.

The anti-inflammatory potential of the studied essential oils was evaluated by an arachidonic acid (AA)-induced edema model in mouse ear. Previous studies have documented the inflammatory response of mouse ears to topical AA, and research indicates that this reaction occurs as a result of AA metabolites being produced through both the cyclooxygenase and lipoxygenase pathways. Arachidonic acid can trigger various events of an inflammatory response, such as an increase in skin thickness and the release of several inflammatory mediators [66]. An increase in skin thickness is among the initial events observed during inflammation, suggesting the presence of edema, epidermal hyperplasia, and increased vascular permeability [46]. In this study, topical applications of AA markedly increased the thickness of the ear skin of mice with respect to the basal state. Conversely, topical treatment with essential oils from *Bursera graveolens*, *Dacryodes peruviana*, *Mespilodaphne quixos*, and *Melaleuca armillaris* reduced this parameter by 25%, 53.3%, 33.42%, and 65.25%, respectively (Figure 3). These results were consistent with the biochemical studies, in which several pro-inflammatory cytokines, including TNF-α, IL-8, IL-17A, and IL-23, were evaluated by Quantitative reverse transcription polymerase chain reaction (RT-qPCR). An increase in the expression of cytokines TNF-α, IL-8, IL-17A, and IL-23 was observed in the positive control. However, this inflammatory response was counteracted by topical treatment of the studied essential oils, mainly *Dacryodes peruviana* and *Melaleuca armillaris*, which caused a decrease in the production of all studied cytokines (Figure 4A–D). On the other hand, *Bursera graveolens* essential oil reduced the expression of IL-8, IL-17A, and IL-23 (Figure 4B–D), whereas *Mespilodaphne quixos* essential oil reduced the expression of IL-17A and IL-23 (Figure 4C,D).

The anti-inflammatory activity exhibited that these essential oils may be due to the major compounds present in them. Specifically, limonene, α-phellandrene, (*E*)-methyl cinnamate, and 1,8-cineole have been reported in previous studies for their role in regulating the inflammatory process. Limonene has been suggested as a potential inductor for the production of the anti-inflammatory cytokine IL-10 and reduction in the expression of TNF-α, IL-1β, and IL-6 [67,68]. Studies have demonstrated the anti-inflammatory effects of limonene in colitis, dermatitis, and lung inflammation caused by allergies [69,70]. α-phellandrene inhibits neutrophil migration toward the inflamed area and reduces cytokines expression induced by TNF-α. Some studies suggest the benefit of α-Phellandrene in inflammatory diseases, such as rheumatoid arthritis, osteoarthritis, and allergic conditions [71]. Regarding methyl cinnamate, reports of its anti-inflammatory activity are scarce, although potential anti-inflammatory applications against periodontal disease and related systemic conditions have been suggested [72]. Finally, published research has documented that the monoterpene 1,8-Cineol reduces the expression of cytokines such as IL-4 and IL-6 in bronchial epithelial cells, as well as influences immune cells, where decreased expression in TNF-α, IL-1β, and IL-6 from monocytes, and also decreased the expression of IL-4 and IL-5 from lymphocytes. Even in acne, 1,8-Cineol-containing leaf extracts suppress the expression of IL-1β and IL-6 [73]. The large number of scientific publications that support the anti-inflammatory potential of α-phellandrene and 1,8-Cineol in the regulation of the inflammatory process and their promising application in the treatment of different inflammatory diseases agree with the results obtained in this investigation, in which the two essential oils that showed greater efficacy correspond to the species *Dacryodes peruviana* and *Melaleuca armillaris*, which are mainly composed of these two components. Therefore, these results suggest that the studied essential oils could be used as alternative therapy or adjuvant to increase the efficacy of conventional therapies in the treatment of dermal inflammation.

## 4. Materials and Methods

### 4.1. Materials

Helium was purchased from INDURA (Quito, Ecuador). The standard aliphatic hydrocarbons (C_9_–C_25_) were purchased from ChemService (West Chester, PA, USA). Anhydrous sodium sulfate and dichloromethane (DCM) were purchased from Sigma-Aldrich (San Luis, MO, USA). All chemicals were of analytical grade and used without further purification. Amphotericin B was obtained from Acofarma (Barcelona, Spain), RPMI-1640 medium, MOPS, glucose, and chloramphenicol were obtained from Sigma-Aldrish (Darmstadt, Germany). Three yeast species were used for the assay: *Candida albicans* ATCC 10231, *Candida glabrata* ATCC 66032, and *Candida parapsilosis* ATCC 22019.

### 4.2. Plant Material

The information on the collection of the four species is shown in Table 4. The species *Mespilodaphne quixos* and *Melaleuca armillaris* were collected in the vegetative stage. Three samples were collected. After being collected, the plant material was stored and transferred in airtight plastic containers (without refrigeration). The botanical specimens were identified by Dr. Nixón Cumbicus at the herbarium of the Universidad Técnica Particular de Loja (HUTPL). A voucher specimen is preserved in the HUTPL.

### 4.3. Essential Oil Isolation

The plant material of Bursera graveolens (10 Kg), Dacryodes peruviana (9 kg), Mespilodaphne quixos (15 Kg), and Melaleuca armillaris (18 Kg) were processed fresh immediately after arriving at the laboratory, between 2 and 6 h after being collected. Extraction of the essential oil was carried out by hydrodistillation in Clevenger-type apparatus (80 L distiller). Initially, 16 L of water was placed in the distiller, then the plant material and the extraction process began [74]. The process was maintained for 3 h. The essential oil was separated from the water by decantation, dried using anhydrous sodium sulfate, and stored at 4 °C until being used in analysis. Each plant species was distilled three times.

### 4.4. Determination of Physical Properties of Essential Oil

The density of the essential oils was determined using the ISO 279:1998 standard [75] (equivalent to the AFNOR NF T 75-111 standard) using an analytical balance (Mettler AC 100, Mettler Toledo, Columbus, OH, USA). The refractive index was determined using the standard ISO 280:1998 [76] (similarly to AFNOR NF T 75-112) using a refractometer (model ABBE, BOECO, Hamburg, Germany). The optical rotation of essential oils was determined according to the standard ISO 592:1998 [77] using an automatic polarimeter (Mrc-P810, MRC, Holon, Israel). The procedures were carried out in triplicate, and all measurements were performed at 20 °C.

### 4.5. Essential Oil Compounds Identification

#### 4.5.1. Quantitative Analysis

The quantitative analysis was performed using gas chromatography coupled to flame ionization detector (GC-FID) for which an Agilent gas chromatograph (GC) (6890N series), a flame ionization detector (FID), a nonpolar GC column (DB-5ms, stationary phase 5%-phenyl-methylpolyxilosane, 30 m of length, 0.25 mm of diameter, and 0.25 µm of stationary layer thickness), and an automatic injector (7683 automatic liquid sampler) were used (all equipment was from Agilent Technologies, Santa Clara, CA, USA). The procedures were carried out as described by Valarezo et al. [49]. Briefly, 1 µL of solution (1/100, *v*/*v*, EO/DCM) was injected with a split ratio of 1:50. Helium was used as a carrier gas at 1 mL/min in constant flow mode and an average velocity of 25 cm/s. The injector and detector temperatures were 250 °C. The oven temperature program included an initial isotherm of 50 °C for 3 min, followed by a temperature ramp to 260 °C at 3 °C/min, and a final isotherm for 3 min. The relative amounts of the compounds were calculated based on the GC peak area (FID response) without using a correction factor.

#### 4.5.2. Qualitative Analysis

The quantitative analysis was performed using gas chromatography coupled to mass spectrometry (GC-MS) for which the same equipment as in the quantitative analysis was used, except for the detector that was replaced by a mass spectrometer (MS) (quadrupole) detector (series 5973 inert, Agilent Technologies, Santa Clara, CA, USA). The procedures were carried out as described by Valarezo et al. [49]. The sample concentration and temperatures (ramp, injector, and detector) were the same as for qualitative analyses. Helium was used as a carrier gas at 0.9 mL/min in constant flow mode and an average velocity of 34 cm/s. For the identification of the compounds, the retention index IR and the mass spectra were compared with published data [43,78].

### 4.6. Cytotoxicity Studies by Methylthiazolyldiphenyl-Tetrazolium Bromide (MTT) Method

An in vitro MTT cytotoxicity assay was conducted using the HaCaT human keratinocyte cell line. The cells were adjusted to a concentration of 2 × 10^5^ cells/mL and seeded in a 96-well plate for 24 h. They were cultured using Dubelcco’s Modified Eagle’s Medium (DMEM), which contained a high glucose level and buffered with 25 mM HEPES (4-(2-hydroxyethyl)-1-piperazineethanesulfonic acid). The medium was supplemented with 1% non-essential amino acids, 100 U/mL penicillin, 100 μg/mL streptomycin, and 10% heat-inactivated Fetal Bovine Serum (FBS). Following the incubation period, the cells were exposed to various dilutions of the essential oils from 500 to 5000 μg/mL using the culture medium as a dilution agent for 24 h. Subsequently, the HaCaT cells underwent a gentle washing process using 1% sterile PBS, followed by incubation with a solution of MTT (Sigma-Aldrich Chemical Co, St. Louis, MO, USA) at a concentration of 2.5 mg/mL for 2 h at 37 °C. After carefully removing the medium, a solubilization reagent (99% DMSO) was added in a volume of 0.1 mL to facilitate cell lysis and dissolve the purple MTT crystals. Cell viability was assessed at a wavelength of 570 nm using a microplate photometer, Varioskan TM LUX (Thermo Scientific, Waltham, MA, USA). In order to make a comparative analysis, a negative control (untreated HaCaT cells) was concurrently processed using the same method. This experiment was carried out in triplicate. The results were expressed as a percentage of cell survival relative to the negative control (100% viability) using Equation (1):

(1) $$\%\mathrm{Cell}\;\mathrm{viability}=\frac{\mathrm{Abs}\;\mathrm{sample}}{\mathrm{Abs}\;\mathrm{control}}\times 100$$

### 4.7. In Vivo Tolerance Studies

Transepidermal water loss (TEWL) measurements were conducted on the ventral area of the forearm in 10 volunteers (aged between 20 and 40 years) who had healthy skin. The participants provided their written informed consent prior to the study. The research protocol received approval from the Ethics Committee of the University of Barcelona (IRB00003099), adhering to the principles of the Declaration of Helsinki. Each essential oil from the four species was diluted to 5% using 55% Transcutol-P and 40% water. TEWL values were determined at basal state and 10 min, 1 h, and 2 h after skin application of essential oils using a Tewameter TM HEX (Courage & Khazaka Electronics GmbH, Cologne, Germany). The probe was pressed on the skin for 20 s, and the results are presented in units of g/cm^2^/h.

### 4.8. Antifungal Efficacy Studies

#### 4.8.1. Preparation of Culture Medium

A total of 5.20 g of RPMI 1640 medium powder was dissolved in 150 mL of distilled water. Subsequently, 17.26 g of MOPS buffer and 9 g of glucose were added until 250 mL was obtained. The culture medium was adjusted to pH 7.0 with 0.1 M NaOH, and 500 µg/mL chloramphenicol was added and passed through a 0.22-micron filter.

#### 4.8.2. Inoculum Preparation

The Candida strains were plated on Sabouraud agar for 48 h at 30 °C. A second replanting of 24 h was then carried out. Isolated colonies (approximately four) were resuspended in Ringer’s solution by vortexing to about 0.5 McFarland turbidity. A standard was prepared separately by adding 0.5 mL of solution A (1.175% *w*/*v* BaCl_2_.2H_2_O) and 99.50 mL of solution B (1% H_2_SO_4_), which were taken to a spectrophotometer and measured at 530 nm. The absorbance should be between 0.120 and 0.150. The colony suspension was adjusted by performing a 1/10 dilution with Ringer’s medium until obtaining an absorbance similar to our standard. The final suspension would be close to 0.5–2.5 × 10^5^ CFU/mL. Assays were as in *EUCAST Assays Definitive Document EDef 7.1: A Method for Determination of Broth Dilution MICs of Antifungal Agents for Fermentative Yeasts* [79].

#### 4.8.3. Antifungal Activity

In a 96-well plate, 100 µL of the culture medium (mentioned in Section 4.8.1) was added (in each well). Subsequently, in the first well of the first two rows (vertically), a previously dissolved solution of Amphotericin B was added to a final concentration of 150 µg/mL (this drug was used as standard). In the following wells (first well vertically), the samples to be tested were added: *Bursera graveolens*, *Dacryodes peruviana*, *Mespilodaphne quixos*, and *Melaleuca armillaris* essential oils (dissolved at 5% with 55% Transcutol P and 40% water), and serial doubling dilutions were made (horizontally). Finally, 100 µL of the inoculum was added to all the wells. The culture medium without inoculum and without drug was used as blank, and the inoculum was used as the negative control. MIC was determined by spectrophotometry using a model 680 microplate reader spectrophotometer (Bio-Rad, Madrid, Spain) at a wavelength of 620 nm. The plates were read at t0 and then at 24 h and 48 h after incubation at 30 °C [79].

### 4.9. In Vivo Anti-Inflammatory Activity: Arachidonic Acid (AA)-Induced Edema

#### 4.9.1. Study Protocol

In vivo experiments were conducted in accordance with the ethical protocol approved by the Bioethics Committee of the University of Barcelona (CEEA/UB ref. 4/16 and Generalitat ref. 8756. Date: 28 January 2016). Male BALB/c mice aged 4–5 months old were used for the study. A solution of 60 μL of AA at 5 mg/mL diluted in PBS was topically applied to the right ears to induce inflammation. Ear thickness was measured before and 15 after the induction of inflammation using a micrometer. A group of inflamed animals (*n* = 3) served as a positive control, whereas a group of untreated mice (*n* = 3) was used as a negative group to compare the results. After 20 min of inducing inflammation, four groups of animals (*n* = 3) were topically treated with 60 μL of *Bursera graveolens*, *Dacryodes peruviana*, *Mespilodaphne quixos*, and *Melaleuca armillaris* essential oils (dissolved at 5% with 55% Transcutol P and 40% water), respectively. In parallel, a group (*n* = 3) was treated with a commercial anti-inflammatory product (60 mg of ibuprofen gel 50 mg/g; reference: 886,192.7), and a last group (referred to as the Blank group) was treated with Transcutol-P:water (60:40, *v*/*v*). After 20 min of these treatments, ear thickness was again measured, and the animals were euthanized by cervical dislocation. Oedema was expressed as the difference between the basal ear thickness and the ear thickness after AA application. The inhibition of inflammation percentage or anti-inflammatory efficacy was calculated based on the decrease in ear thickness after each treatment. Finally, the right ears were cut to evaluate the expression of pro-inflammatory cytokines.

#### 4.9.2. Time Quantitative PCR to Assay Inflammatory Biomarkers

Small portions of mouse ear tissue were disrupted using a Bead Beater homogenizer (MP Biomedicals, Madrid, Spain) and the TRI-Reagent^®^ solution (Sigma Aldrich, St. Louis, MO, USA), a phenol-based reagent (named as TRIzol). Total RNA was extracted following the manufacturer’s protocol. Integrity and total amount of RNA were tested by NanoDrop One spectrophotometer (Thermo Scientific, Waltham, MA, USA). Then, 500 ng of RNA was retrotranscribed into complementary DNA using the High-Capacity cDNA Reverse Transcription Kit (Applied Biosystems, Foster City, CA, USA). Finally, a dilution of 1/10 of cDNA was used to perform the RT-qPCR. Therefore, the SYBR Green PCR Master Mix (Applied Biosystems, Foster City, CA, USA) and specific pair of primers for inflammatory genes (IL-8, IL-17A, IL-23, and TNFα) were added to the reaction. As a housekeeping gene, the GAPDH was selected and used for normalization. Relative changes in gene expression were analyzed by applying the 2^−ΔΔCt^ method. Table 5 shows the primers used for the Real-Time qPCR.

### 4.10. Statistical Analysis

The results were presented as mean ± SD. Statistical analysis was carried out using GraphPad Prism, version 6.0 software (GraphPad Software Inc., San Diego, CA, USA). One-way ANOVA, followed by Tukey’s test, was used to compare the mean values. Statistical significance was set at a *p* value less than 0.05.

## 5. Conclusions

In conclusion, this study demonstrates through its different experiments that the essential oils obtained from *Bursera graveolens*, *Dacryodes peruviana*, *Mespilodaphne quixos*, and *Melaleuca armillaris* can be topically used in dermal treatments as they exhibited high biocompatibility with human keratinocytes as well as an occlusive effect on the skin of healthy volunteers, improving hydration without causing irritation or damage to the integrity of the stratum corneum. Additionally, these essential oils showed antifungal activity against *Candida albicans*, *Candida glabrata*, and *Candida parapsilosis*, possibly thanks to their rich content in limonene, α-phellandrene, (*E*)-methyl cinnamate, and 1,8-cineole, respectively. Similarly, these essential oils effectively counteracted and alleviated two critical events in dermal inflammatory processes, such as edema and the overexpression of pro-inflammatory cytokines, including TNF-α, IL-8, IL-17A, and IL-23. The pharmacological potential of these essential oils suggests that they could be used both as active antifungal and anti-inflammatory components or adjuvants to increase the efficacy of conventional therapies in the treatment of both cutaneous candidiasis and dermal inflammation.

## Figures and Tables

**Table 1:** Yield and physical properties of the essential oils from *Bursera graveolens* (BGR), *Dacryodes peruviana* (DPE), *Mespilodaphne quixos* (MQU), and *Melaleuca armillaris* (MAR).

	BGR		DPE		MQU		MAR	
	Mean	SD	Mean	SD	Mean	SD	Mean	SD
Yield (mL/kg)	31	2	45	3	15	1	9	1
Density, ρ (g/cm^3^)	0.8385	0.0010	0.8456	0.0023	0.8758	0.0013	0.9053	0.0045
Refractive index, n20	1.4760	0.0011	1.4751	0.0002	1.4790	0.0012	1.4641	0.0023
Specific rotation, [α] (°)	47.7	1.1	12.2	0.7	11.2	0.2	4.3	1.1

**Table 2:** Majority compounds (>1%) of the essential oils from *Bursera graveolens* (BGR), *Dacryodes peruviana* (DPE), *Mespilodaphne quixos* (MQU), and *Melaleuca armillaris* (MAR).

Compounds	RIC	RIR	BGR		DPE		MQU		MAR		Type
			%	SD	%	SD	%	SD	%	SD	
α-Thujene	926	924	-		1.9	0.1	-		-		MH
α-Pinene	932	932	-		8.3	0.3	1.6	0.1	3.6	0.1	MH
Sabinene	969	969	-		1.4	0.4	-		-		MH
β-Pinene	973	974	-		2.6	0.9	1.3	0.1	1.4	0.1	MH
Myrcene	986	988	1.1	0.4	-		-		1.3	0.1	MH
α-Phellandrene	1005	1002	35.9	1.3	50.3	3.3	-		-		MH
*p*-Cymene	1021	1020	-		3.1	0.8	-		-		MH
Limonene	1025	1024	49.4	2.2	23.0	1.5	-		-		MH
1,8-Cineole	1025	1026	-		-		1.3	0.1	83.4	2.5	OM
Terpinolene	1082	1086	-		5.2	0.9	-		-		MH
Menthofuran	1159	1159	6.6	1.2	-		-		-		OM
α-Terpineol	1187	1186	-		-		-		8.0	0.1	OM
(*E*)-Cinnamaldehyde	1268	1267	-		-		10.0	1.4	-		PP
α-Copaene	1372	1374	-		-		4.5	0.9	-		SH
(*E*)-Methyl cinnamate	1376	1376	-		-		19.3	1.3	-		PP
*(E)*-Caryophyllene	1415	1417	-		-		15.8	1.8	-		SH
6,9-Guaiadiene	1442	1442	-		-		4.5	0.5	-		SH
(*E*)-Cinnamyl acetate	1445	1443	-		-		12.5	0.7	-		PP
Germacrene D	1476	1480	1.5	0.1	-		-		-		SH
β-Selinene	1489	1489	-		-		5.8	0.5	-		SH
Bicyclogermacrene	1496	1500	-		-		4.2	0.4	-		SH
Anisyl propanoate	1510	1511	-		-		2.7	0.4	-		OC
7-*epi*-α-Selinene	1520	1520	-		-		2.5	0.3	-		SH
(*E*)-γ-Bisabolene	1527	1529	-		-		3.1	0.3	-		SH
Caryophyllene oxide	1580	1582	-		-		2.5	0.4	-		OS
Monoterpene hydrocarbons (MH)			86.4		95.8		3.0		6.2		
Oxygenated monoterpene (OM)			6.6		-		1.3		91.4		
Sesquiterpene hydrocarbons (SH)			1.5		-		40.4		-		
Oxygenated sesquiterpene (OS)			-		-		2.5		-		
Phenylpropanoids (PP)			-		-		41.8				
Other compounds (OC)			-		-		2.7		-		
Total identified			94.6		95.8		91.7		97.6		

RIC: calculated retention rates; RIR: reference retention indices, Adams, R.P. (2007) [43]; %: mean percentage obtained from 9 replicates; SD: standard deviation.

**Table 3:** Antifungal activity of essential oils from *Bursera graveolens* (BGR), *Dacryodes peruviana* (DPE), *Mespilodaphne quixos* (MQU), and *Melaleuca armillaris* (MAR).

	MIC (µg/mL)		
	*Candida albicans*	*Candida glabrata*	*Candida parapsilosis*
Amphotericin B	0.15	0.60	0.30
BGR	524.06	262.03	524.06
DPE	132.13	32.98	65.96
MQU	273.69	273.69	136.84
MAR	565.81	565.81	565.81
Blank	-	-	-

**Table 4:** Location and collection conditions of plant species.

Species	Plant Part	Ambient Conditions		Parish	Canton	Province	Coordinates		Altitude (m a.s.l.)
		T (°C)	P (atm)				Latitude	Longitude	
*Bursera graveolens*	Fruits	26	0.98	Garza Real	Zapotillo	Loja	4°19′13” S	80°17′58” W	150
*Dacryodes peruviana*	Fruits	25	0.89	La Paz	Yacuambi	Zamora Chinchipe	3°40′13” S	78°54′21” W	1025
*Mespilodaphne quixos*	Leaves	24	0.91	Pano	Tena	Napo	1°01′12” S	77°51′57” W	650
*Melaleuca armillaris*	Leaves	29	0.79	Guayllabamba	Quito	Pichincha	0°04′43” S	78°20′59” W	2171

T: temperature; P: pressure; a.s.l: above sea level.

**Table 5:** Gene-specific primers used in the Real-Time qPCR.

Gene	Primer Sequence (5′ to 3′)
GAPDH	FW: AGCTTGTCATCAACGGGAAG
	RV: TTTGATGTTAGTGGGGTCTCG
IL-8	FW: GCTGTGACCCTCTCTGTGAAG
	RV: CAAACTCCATCTTGTTGTGTC
IL-23	FW: GAGCCTTCTCTGCTCCCTGATA
	RV: GACTGAGGCTTGGAATCTGCTG
IL-17A	FW: TTTTCAGCAAGGAATGTGGA
	RV: TTCATTGTGGAGGGCAGAC
TNFα	FW: AACTAGTGGTGCCAGCCGAT
	RV: CTTCACAGAGCAATGACTCC

GAPDH = Glyceraldehyde-3-Phosphate Dehydrogenase; IL-8 = interleukin-8; IL-23 = interleukin-23; IL-17A = interleukin-17A; TNFα = Tumor necrosis factor alpha; FW = forward primer; and RV = reverse primer.

## Data Availability

Data are contained within the article.
